# The first complete mitochondrial genome of Dorippoidea from *Orithyia sinica* (Decapoda: Orithyiidae)

**DOI:** 10.1080/23802359.2018.1467237

**Published:** 2018-04-28

**Authors:** Shengping Zhong, Yanfei Zhao, Qin Zhang

**Affiliations:** Key Laboratory of Marine Biotechnology, Guangxi Institute of Oceanology, Beihai, China

**Keywords:** Mitochondrial genome, *Orithyia sinica*, Decapoda

## Abstract

The tiger crab Orithyiidae is the monophyletic family of Dorippoidea. However, the systemic classification and phylogenetic studies have so far been limited. In this study, we report the first complete mitochondrial genome of Dorippoidea from *Orithyia sinica*. The mitogenome has 15,568 base pairs (69.5% A + T content) and is made up of a total of 37 genes (13 protein-coding, 22 transfer RNAs and 2 ribosomal RNAs), and a control region. This is the first available study on complete mitogenomes of Orithyiidae and will provide useful genetic information for future phylogenetic and evolutionary classification of Orithyiidae.

The tiger crab Orithyiidae contains only a single genus and species, which is found in the seas and shallow waters on sandy or muddy substrates of China, Korea and Japan (Schweitzer and Feldmann 2000). The Orithyiidae traditionally belonged to the family Calappidae, which had been reconsidered as the possible sister group of the family Dorippidae and revised as the new family belonging to superfamily Dorippoidea (Bellwood 1996). There are various species belonging to the Dorippoidea, however, in spite of its evolutionary and ecological importance, adequate genetic information about the Dorippidae is still missing (Sin et al. 2009). Here, we report the first complete mitochondrial genome sequence of superfamily Dorippoidea, to provide a better insight into the phylogenetic assessment and evolutionary classification.

Tissue samples of *O. sinica* from five individuals were collected from GuangXi province, China (Beihai, 21.520195 N, 109.158585 E), and the whole body specimen (#GQ0215) were deposited at Marine biological Herbarium, Guangxi Institute of Oceanology, Beihai, China. The total genomic DNA was extracted from the muscle of the specimens using an SQ Tissue DNA Kit (OMEGA, Guangzhou, China) following the manufacturer’s protocol. DNA libraries (350 bp insert) were constructed with the TruSeq NanoTM kit (Illumina, San Diego, CA) and were sequenced (2 × 150 bp paired-end) using HiSeq platform at Novogene Company, China. Mitogenome assembly was performed by MITObim (Hahn et al. 2013). The cytochrome oxidase subunit 1 (COI) gene of *O. sinica* (GenBank accession number: KF364344) was chosen as the initial reference sequence for MITObim assembly. Gene annotation was performed by MITOS (Bernt et al. 2013).

The complete mitogenome of *O. sinica* was 15,568 bp in length (GenBank accession number: MG840649), and consisted 37 genes in total, the typical set of 13 protein-coding genes, 22 tRNA and 2 rRNA genes, and a putative control region. The overall base composition of the mitogenome was estimated to be A 34.6%, T 34.9%, C 19.4%, and G 11.1%, with a high A + T content of 69.5%, which is similar, but slightly lower than *Scylla paramamosain* (72.9%) (Zhong et al. 2017). The result of phylogenetic tree of 17 species (including other 16 species from subsection Heterotremata in NCBI) indicated the close relationship among Dorippoidea, Bythograeoidea, and Xanthoidea (Figure 1), which is different from the phylogenetic analyses of subsection Heterotremata using sequence data of six nuclear protein-coding genes and two mitochondrial rRNA genes (Tsang et al. 2014). Further taxonomic studies for Heterotremata are needed in future work. The complete mitochondrial genome sequence of *O. sinica* was the first sequenced mitogenome in Dorippoidea, which will contribute to further phylogenetic and comparative mitogenome studies of Dorippoidea, and related families.

**Figure 1.**
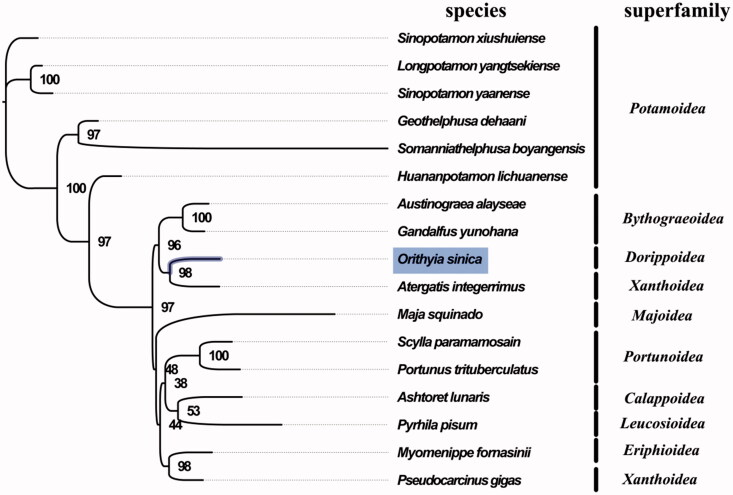
Phylogenetic tree of 17 species in subsection Heterotremata. The complete mitogenomes is downloaded from GenBank and the phylogenic tree is constructed by maximum-likelihood method with 100 bootstrap replicates. The bootstrap values were labelled at each branch nodes. The gene's accession number for tree construction is listed as follows: *Sinopotamon xiushuiense* (NC_029226), *Longpotamon yangtsekiense* (NC_036946), *Sinopotamon yaanense* (NC_036947), *Geothelphusa dehaani* (NC_007379), *Somanniathelphusa boyangensis* (NC_032044), *Huananpotamon lichuanense* (NC_031406), *Austinograea alayseae* (NC_020314), *Gandalfus yunohana* (NC_013713), *Atergatis integerrimus* (NC_037172), *Maja squinado* (NC_035425), *Scylla paramamosain* (NC_012572), *Portunus trituberculatus* (NC_005037), *Ashtoret lunaris* (NC_024435), *Pyrhila pisum* (NC_030047), *Myomenippe fornasinii* (NC_024437), and *Pseudocarcinus gigas* (NC_006891).
